# Design and Evaluation of Multi-Electrode Configurations for Transcutaneous Frontal Nerve Activation

**DOI:** 10.3390/bios16090519

**Published:** 2026-09-17

**Authors:** Enver Salkim

**Affiliations:** 1 Department of Electronic and Automation, Muş Alparslan University, Muş 49250, Türkiye; e.salkim@alparslan.edu.tr or e.salkim@ucl.ac.uk; 2 Department of Electronic and Electrical Engineering, University College London, London WC1E 6BT, UK

**Keywords:** computational modelling, electrode array, electrode alignments, neuro-anatomical variations, transcutaneous nerve stimulation

## Abstract

Conventional migraine treatments often come with undesirable side effects. One alternative approach uses a wearable neuromodulation device that stimulates nerves in the forehead. However, clinical studies have reported modest effectiveness, and some users experience discomfort or pain. These issues may be associated with electrode design, which can require higher stimulation currents to achieve activation. This study aims to evaluate the stimulation currents required by the migraine neuromodulator device and to explore whether modifying electrode arrangement can lower these current levels, which may potentially improve patient comfort and treatment compliance. To accomplish this, a hybrid computational framework combining a 3D volume conductor model with biophysical nerve dynamics was deployed. To capture population diversity, forty distinct nerve models were generated based on statistical variations in trajectory paths and evaluated across five electrode array configurations. The findings show that the proposed electrode array (EA) and neuroanatomical variability significantly influence stimulation thresholds. Simulations of the Cefaly electrode produced current thresholds ranging from 9 mA to 30 mA across the models. When the proposed EA was applied by aligning with nerve pathways, the required currents dropped substantially, ranging from 0.5 mA to 9 mA. Additionally, this design reduced calculated peak spatial current density across superficial tissue layers—a factor associated with cutaneous discomfort—which may support improved tolerance and long-term treatment adherence in clinical practice.

## 1. Introduction

Migraine is a neurological condition marked by recurring headaches that can affect one or both sides of the head and are often described as pulsating in nature. These episodes are commonly accompanied by symptoms such as nausea, sensitivity to light, and sensitivity to sound [1]. It ranks as the third most prevalent neurological condition and the sixth leading cause of disability, impacting roughly 18% of people worldwide [2]. This widespread occurrence places a significant socioeconomic strain on societies across the globe [3,4].

Existing drug-based treatments for migraine are often linked to moderate or severe side effects, including the risk of worsening headaches due to medication overuse. As a result, patients may experience reduced effectiveness, dissatisfaction, or discontinue their treatment altogether. Recent research highlights a strong need for alternative therapeutic approaches [5].

Neuromodulation has been used for pain management by influencing peripheral or central nervous system pathways through the application of electrical stimulation, either via implanted electrodes or non-invasive surface electrodes [2]. According to the gate control theory, stimulating peripheral nerves activates Aβ afferent fibres, which in turn suppress the activity of Aδ and C fibres responsible for transmitting pain signals [6]. Pain relief may also result from neural plasticity, the nervous system’s capacity to adapt and reorganize itself developing gradually over weeks or months in response to external stimulation [2].

Neuromodulation techniques, such as invasive stimulation of the occipital and vagus nerves, and sphenopalatine ganglion stimulation, as well as non-invasive methods like vagus and frontal nerve stimulation and transcranial magnetic stimulation (TMS), are used in managing migraines. Invasive methods are generally reserved for patients with migraines that do not respond to other treatments due to their potential risks [7]. TMS induces electrical stimulation in the brain, which can activate a range of neurons, potentially leading to disorganized neural activity [8]. Additionally, vagus nerve stimulation, which targets both sensory and motor fibres of the vagus nerve, may result in neck muscle contractions. Furthermore, there is a lack of extensive controlled trials to conclusively support the use of TMS and vagus nerve stimulation for migraine prevention [9].

**Figure 1:**
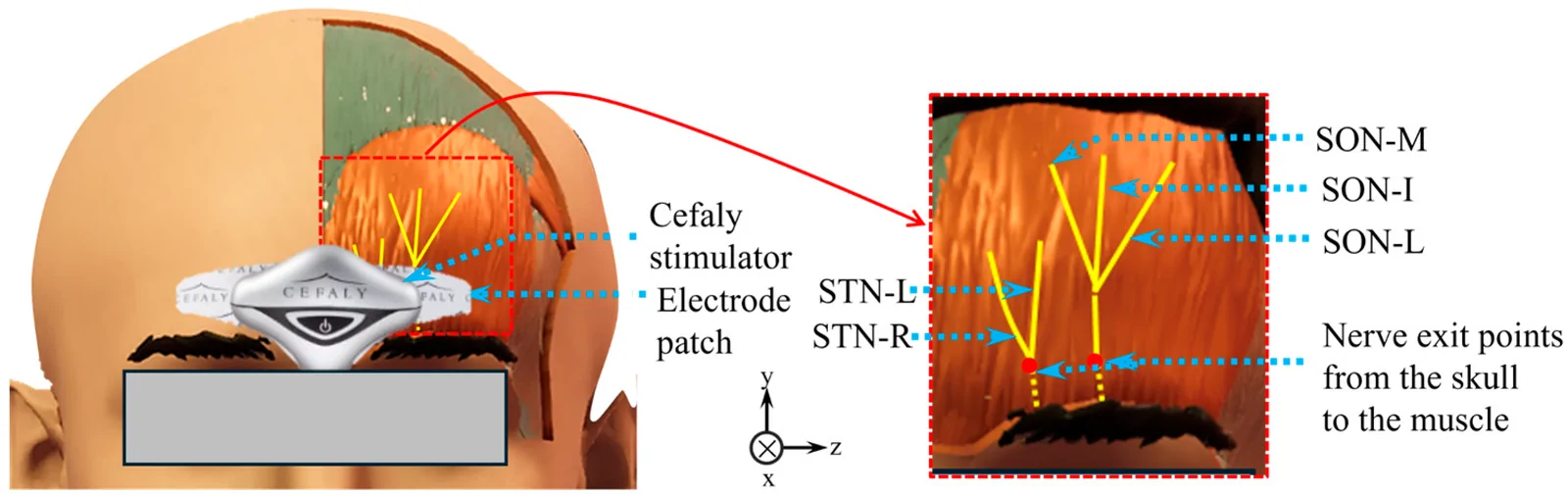
The Cefaly electrode neuromodulation system with electrode configuration and the distribution of target nerve variations in the human forehead region.

Migraine sufferers often report pain localized to areas innervated by the supraorbital (SON) and supratrochlear (STN) nerves, which are branches of the frontal nerve from the ophthalmic division of the trigeminal nerve [10], as shown in Figure 1. These nerves transmit pain signals from the frontal head to the trigeminal nucleus caudalis and then to the thalamus, which relays the information to higher brain centers. Transcutaneous frontal nerve stimulation (t-FNS), particularly with the Cefaly neurostimulator, has been shown to help prevent episodic migraines. This Cefaly device applies biphasic transcutaneous current through flexible, gel-coated metal electrodes placed across the forehead to stimulate the target nerves, as shown in Figure 1. A double-blind randomized controlled trial (n = 67) evaluated the efficacy, tolerability, and safety of the Cefaly device for t-FNS, yielding mixed results with a 50% response rate [11]. A post-marketing survey of 2313 users showed 53% satisfaction, though paraesthesia and painful sensations were reported as the main limitations. These inconsistent findings could be due to neuroanatomical variations, which may require higher current levels to achieve effectiveness [12,13]. Since the Cefaly device is operated by the patient, these high currents are not typically used, potentially leading to insufficient stimulation. Additionally, the placement of the electrodes near pain-sensitive areas, such as the eyes and frontal sinuses, could cause discomfort even at low current levels, further limiting the device’s effectiveness. Also, it has been shown that the effectiveness of the neuromodulator can be varied based on the electrode configurations [14,15]. Although anatomical variations have been shown to significantly influence nerve activation, the effects of employing different electrode arrangements on target nerve branches have not yet been systematically investigated [16].

Despite widespread clinical interest, comprehensive investigations identifying the specific causes of treatment inefficacy in transcutaneous cranial nerve stimulation remain limited. A primary contributing factor is the practical challenge of evaluating neuroanatomical variations across large, statistically representative patient cohorts. To address this limitation, computational modeling allows neuroanatomical parameters to be systematically varied according to statistical distributions derived from experimental anatomical data. In this study, forty statistically generated nerve trajectories were evaluated within an MRI-derived head model to capture population-level anatomical diversity.

The computational framework employed here utilizes a hybrid modeling approach consisting of two primary components: a finite-element volume conductor model and a multi-compartment neural tissue model. The volume conductor model calculates the spatial electric field generated by a given electrode array configuration [16,17]. The resulting extracellular electric potentials along the nerve pathways are then transferred to the multi-compartment neural model to simulate branch-specific fiber excitation and determine activation thresholds.

Previous studies have demonstrated that orienting the primary axis of a Cefaly-like electrode to align with underlying target nerves reduces the stimulus current required for activation; however, certain nerve branches continue to exhibit elevated thresholds [16]. Further threshold reductions can be achieved by optimizing electrode arrangements. Specifically, aligning multi-contact electrode elements along the target nerve path induces favorable potential gradients along the fiber trajectory, thereby lowering the stimulus current required for excitation. To implement this mechanism, the present study evaluates multi-contact circular electrode arrays (EAs) of varying dimensions.

Positioning the proposed EA along the principal trajectory of the nerve exposes a greater portion of the neural pathway to activating current gradients while simultaneously steering current density away from non-target, sensitive tissue layers [18]. This study systematically assesses the extent to which this path-aligned approach lowers required activation thresholds across a statistically representative set of nerve models within an anatomically realistic human head volume conductor.

Building upon prior evidence that aligning electrode elements with underlying nerve trajectories lowers stimulation thresholds in simplified models [16], this study offers a significant advancement in targeted array engineering and dielectric modeling fidelity. All computational simulations were conducted using an anatomically detailed human head model incorporating realistic, layer-specific dielectric properties evaluated across four major target branches: the intraorbital, medial, and lateral branches of the supraorbital nerve (SON-I, SON-M, SON-L) and the supratrochlear nerve (STN). We introduce a multi-contact electrode array specifically structured to trace superficial frontal nerve pathways and evaluate branch-specific activation thresholds ($I_{50}$). Statistical analysis confirms that the proposed EA significantly decreases activation thresholds compared to the commercial Cefaly electrode. Furthermore, spatial current density profiles demonstrate that the proposed array minimizes local electrical hot-spots within pain-sensitive superficial layers, which may improve patient comfort and tolerance, ultimately supporting enhanced therapeutic performance in prospective clinical applications.

The remainder of this paper is structured as follows. Section 2 describes the methodology used to develop the volume conductor model of the human head, including the associated tissue layers and myelinated nerve fibres. Section 3 presents the simulation results, focusing on the effects of neuroanatomical variability on stimulation thresholds using and proposed EA and Cefaly electrode. Section 4 discusses the key findings, and Section 5 provides the conclusions along with directions for future research.

## 2. Methods

### 2.1. Human Head Model Development

**Figure 2:**
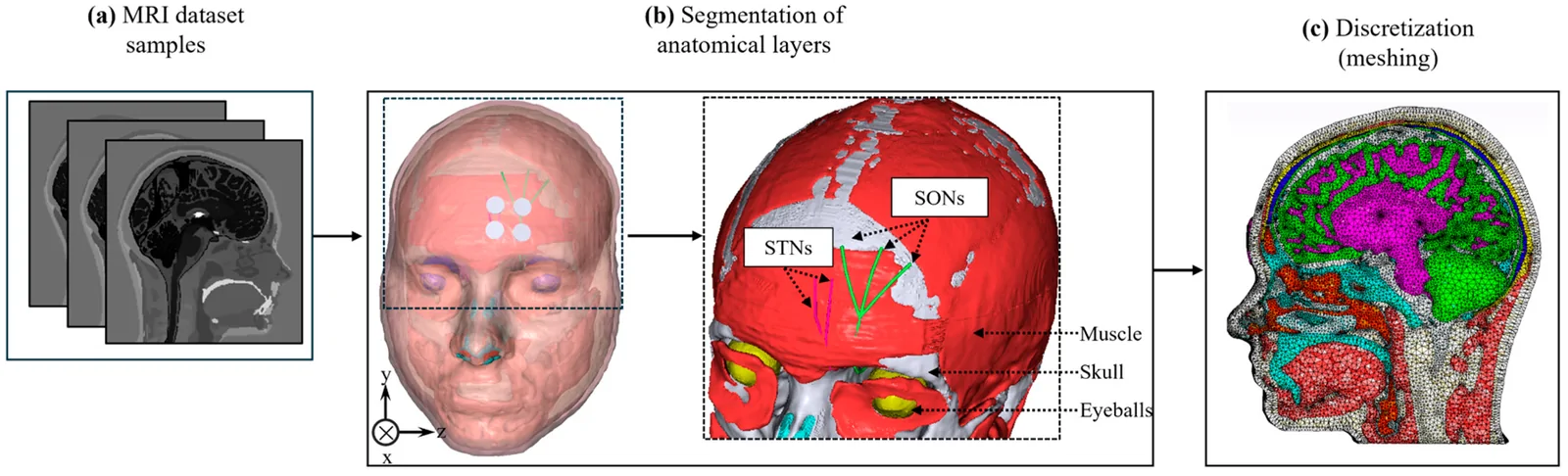
(**a**) Anatomical layers segmented from the imaging dataset. (**b**) Three-dimensional finite element (FE) domains reconstructed from individual image sets using labelling and smoothing operations in ScanIP. Electrode geometry generated and incorporated into the 3D head volume conductor model. (**c**) The assembled model is then discretized and subjected to a current source with appropriate boundary conditions.

A three-dimensional (3D) anatomical model of the human head was derived from a high-resolution MRI dataset (https://itis.swiss/virtual-population/regional-human-models/mida-model, accessed on 9 June 2026) consisting of 350 slices, each with a resolution of 480 × 480 pixels, as shown in Figure 2a. The voxel size was 0.5 × 0.5 × 0.5 mm along the x, y, and z axes [19]. The dataset was imported into Simpleware ScanIP (Synopsys, Mountain View, CA, USA) for image processing and segmentation. The fundamental tissue layers including skin, fat, muscle, eyeball, skull, cerebrospinal fluid (CSF), and brain tissue (gray and white matter) were segmented using a combination of automated and manual techniques. For most tissues, predefined grayscale thresholds enabled automated segmentation, as shown in Figure 2b. However, manual refinement was necessary for certain regions due to discontinuities (e.g., CSF) and overlapping grayscale intensities between adjacent structures. Without such corrections, highly conductive tissues like CSF could significantly alter current distribution within the model [20]. Segmentation accuracy was further improved using smoothing filters (e.g., recursive Gaussian, median, and mean) and morphological operations (such as dilation, erosion, opening, and closing) available in ScanIP. Boolean operations were subsequently applied to eliminate any overlaps between segmented tissue layers.

**Figure 3:**
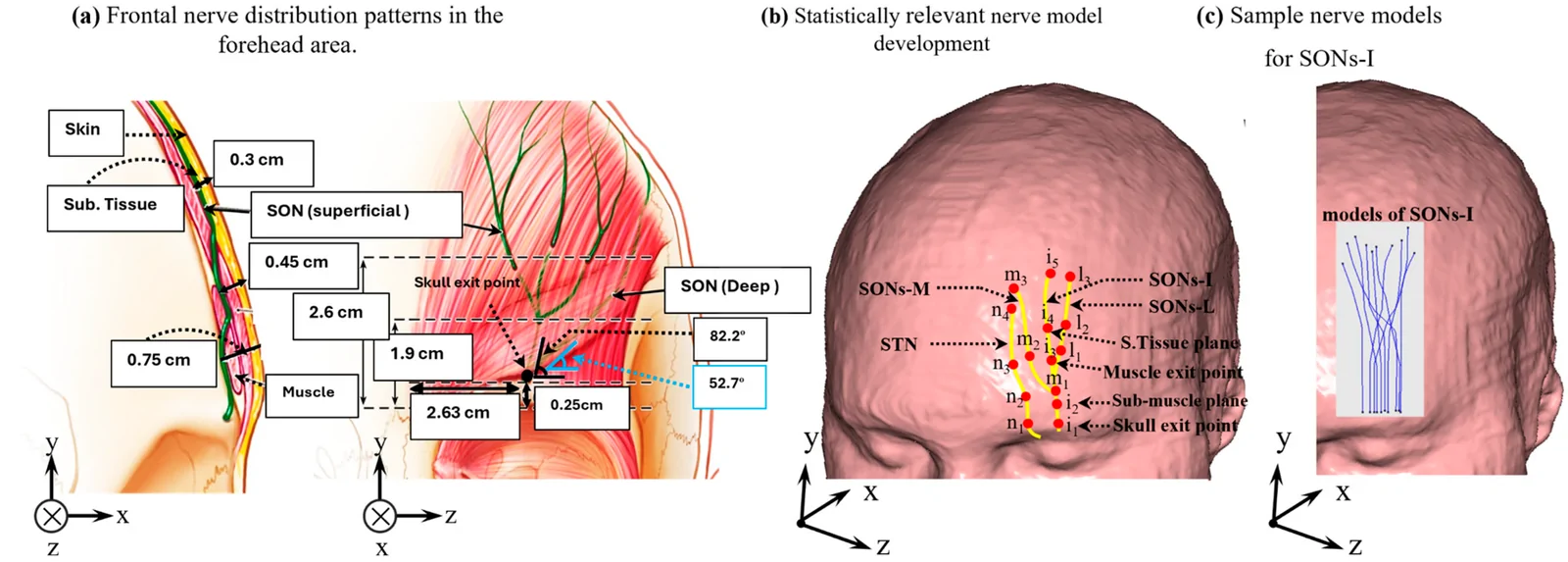
(**a**) Distribution patterns of SON nerve trajectories in the forehead region. (**b**) Example variations of nerve branches in the human head model. All nerve branches are shown in yellow, while branching transition points are indicated by red dots and labelled in black. (**c**) Representative nerve distributions. Ten statistically SONs-I relevant nerve configurations were generated, and distributions are highlighted in blue.

**Figure 4:**
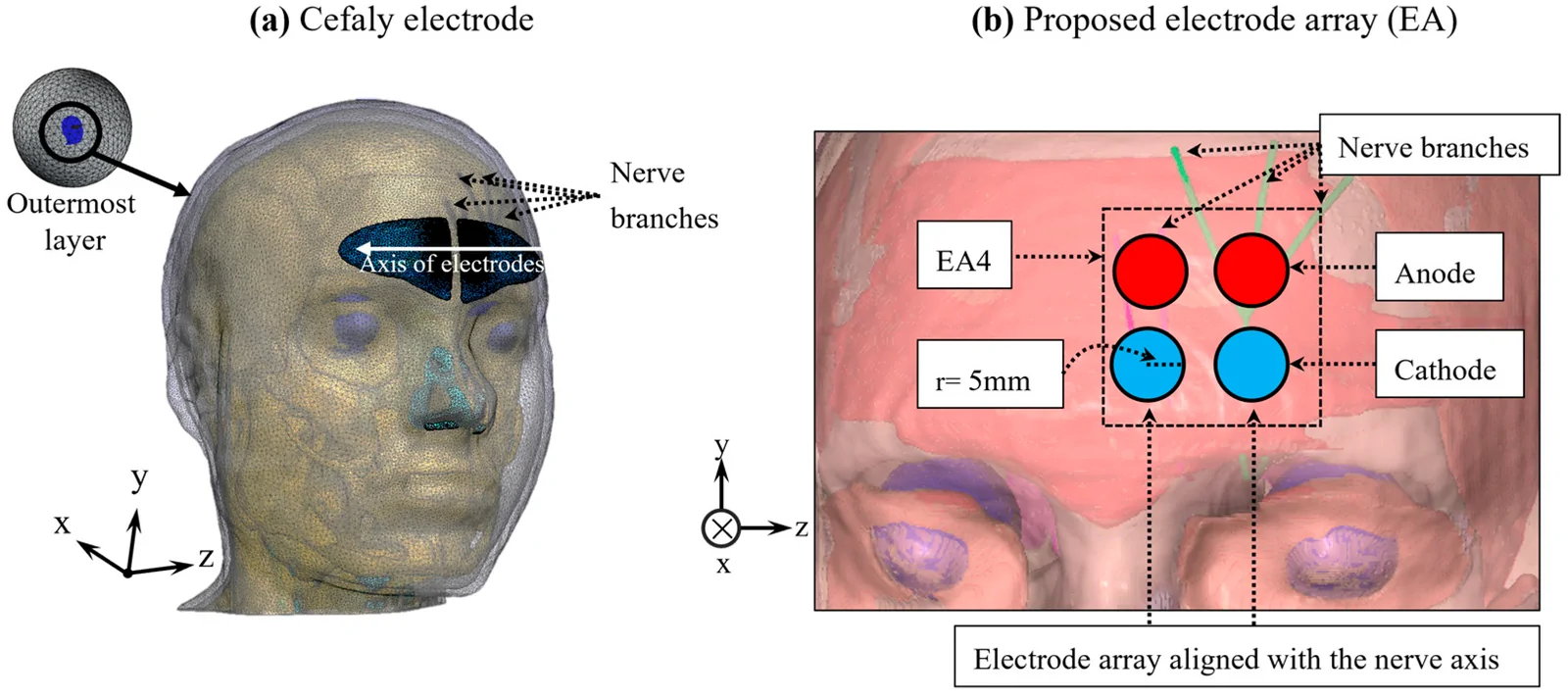
(**a**) The Cefaly electrode is integrated with the human head model for simulation of nerve stimulations. (**b**) The proposed EA integrated with the segmented human head model for subsequent analysis. Nerve branches are highlighted in both cases. EAs are aligned with nerve axis.

Since the SONs and STN have small diameters (approximately 1 mm), their trajectories are not readily visible in MRI data [21]. Therefore, their 3D representations were constructed using simplified geometric primitives (e.g., cylinders) in ScanIP, based on reported statistical anatomical distributions [22,23] as a sample was represented in Figure 3a. These nerve pathways were modelled using multiple cylindrical segments of equal diameter but varying lengths to approximate their course from within the skull to the subcutaneous region. The individual segments were combined using Boolean operations and smoothed to ensure continuous, non-irregular trajectories. Assuming bilateral symmetry of the human head, nerve models were generated only on the left side, as illustrated in Figure 3b. The electrode model was created separately and subsequently integrated with the skin layer for all EAs. A complete set of segmented tissues and the EAs was presented in Figure 4.

### 2.2. Neuroanatomical Variation Design

The supraorbital nerve (SON) emerges at the superior orbital rim and courses beneath the corrugator supercilii muscle (CSM), eventually reaching the plane below the frontalis muscle. This may occur either by directly piercing the CSM or by traveling beneath it before emerging at the muscle’s superior border. As it progresses within the frontalis muscle, the SON typically divides into three primary superficial branches: the medial (SONs-M), intermediate (SONs-I), and lateral (SONs-L), as shown in Figure 3a. For modelling simplicity, finer terminal nerve filaments were not included. These three branches penetrate the frontalis muscle at different locations and follow variable trajectories within the subcutaneous layer, providing sensory innervation to the forehead [22,23]. The STN exits the orbital rim either as a single branch or bifurcates into two branches at that point. It then passes through the CSM and subsequently penetrates the frontalis muscle to enter the subcutaneous plane [24].

Anatomical studies have demonstrated considerable inter-individual variability in the exit points, pathways, and branching patterns of both the SON and STN across different tissue planes. To assess how these variations influence the percentage activation (PA) of nerve fibres under a given stimulus current, such variability must be incorporated into the model. Statistical descriptions of nerve trajectories and exit locations have been well documented in prior anatomical studies [22,23,24]. In this work, the intermediate branch (SONs-I) was treated as the reference or central branch.

As the SON transitions from the skull region into the subcutaneous layer, five key transition points (i_1_ to i_5_, shown in Figure 3b) were defined along the trajectory of the SONs-I. Although the exact branching location varies, it generally occurs within the frontalis muscle. Therefore, the SONs-I was used as a reference to determine the branching locations of the medial and lateral branches. The coordinates of these branching points (m_1_–m_3_ for SONs-M and l_1_–l_3_ for SONs-L, as illustrated in Figure 3b) were derived from reported anatomical data [22,23]. The length of the SON trajectory within the skin and subcutaneous layers was assumed to be 15 mm. For the STN, the anatomical pathways of its branches are largely similar as they traverse from the skull to the subcutaneous region. Previous findings indicate that the stimulus current required for activation is comparable across these branches when evaluated using PA metrics [24]. Accordingly, the STN was modelled as a single branch in this study. Its trajectory and corresponding transition points (n_1_ to n_4_) are depicted in Figure 3b.

To ensure physiologically realistic and smooth nerve geometries while avoiding irregular or zigzag trajectories, three criteria (equations) were imposed.

(1) $$\forall (i_{\left(k+1\right)}^{z}-i_{k}^{z})<0$$

(2) $$\forall (i_{\left(k+1\right)}^{z}-i_{k}^{z})>0$$

(3) $$i_{1}^{y}<i_{2}^{y}<i_{3}^{y}<i_{4}^{y}<i_{5}^{y}$$

where *i* denotes a point along the nerve trajectory, while *k* represents the corresponding transition point index. Equations (1) and (2) were applied to the first two points of all nerve models. If the difference between these initial points was negative, all subsequent points were required to satisfy Equation (1). Conversely, if the difference was positive, the remaining points had to conform to Equation (2). To ensure that the nerve path originates at the orbital rim and extends through the superior region of the forehead, Equation (3) was additionally enforced. Equation (1) enforces a trajectory that begins at the orbital rim and progresses toward the central forehead region along the z-direction. In contrast, Equation (2) ensures that the nerve deviates away from the midline in the z-direction. Equation (3) governs the incremental progression of the nerve along the y-direction. Since the nerve paths are expected to include curvature, consecutive identical points (i.e., $i_{k+1}^{z}-i_{k}^{z}=0$) were excluded. These constraints were first applied to the SONs-I branch. The same set of criteria and conditions were subsequently extended to all SON branches as well as the STN in this study.

The nerve models were defined according to their statistical distributions, while the nerve transition points were generated randomly using an automated variable-generation script in MATLAB v2023. This procedure was repeated sequentially for all model realizations.

After each nerve trajectory was created, minimum separation constraints were enforced between successive points, requiring differences of at least 1.25 mm in the x-direction, 1.5 mm in the z-direction, and 10 mm in the y-direction. For each of the SON branches and the STN, 10 distinct variations were generated, resulting in a total of 40 statistically representative nerve trajectories, with example realizations shown in Figure 3c. Finally, the transition points of each trajectory were interpolated in MATLAB to produce smooth yet anatomically plausible nerve paths.

This study was designed as a deterministic computational proof-of-concept to evaluate the spatial field alignment of path-aligned electrode arrays against conventional geometry. To eliminate confounding inter-subject variance (such as individual differences in skull thickness or subcutaneous fat layers), simulations were conducted within a single, high-fidelity MRI-derived head model. Stochastic nerve trajectories (N = 10 per branch) were utilized to model localized anatomical trajectory dispersion rather than population-level variance. Comparative performance is evaluated via absolute threshold differences ($\Delta I_{50}$) and percentage reductions rather than population inferential statistics.

### 2.3. Electrode Array Design

**Figure 5:**
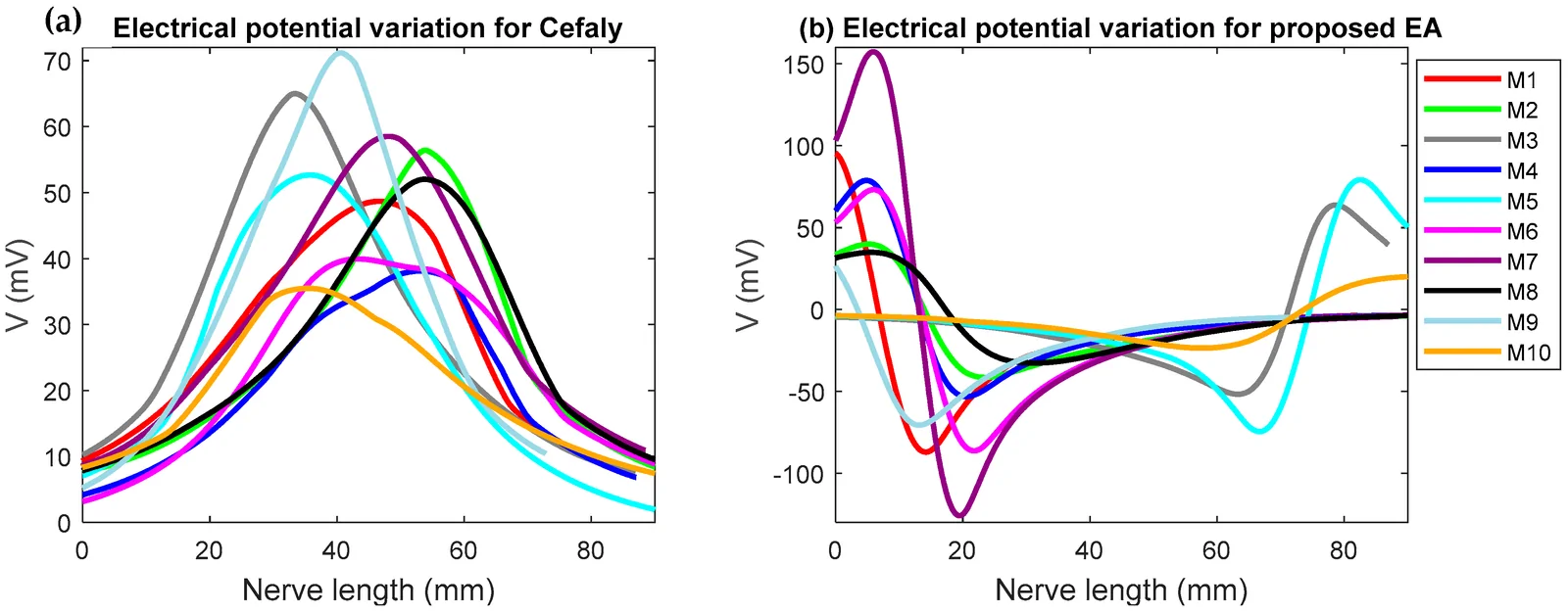
Extracellular potentials variation along the nerve trajectory for (**a**) Cefaly electrode and (**b**) proposed electrode array based on ten SONs-I models M1 through M10.

A key design mechanism of the proposed multi-contact EA is aligning the electrode axis with target nerve trajectories to induce spatial polarity reversals in the extracellular potential along the nerve pathway. The commercial Cefaly electrode [11] and the proposed EA configurations are illustrated in Figure 4a and Figure 4b, respectively. Extracellular potential profiles generated by both electrode layouts were simulated and evaluated across ten representative SON-I nerve trajectory models (Figure 5). Compared to the single-polarity gradient produced by the Cefaly electrode (Figure 5a), the proposed EA generates multiple spatial potential oscillations along the nerve fibers (Figure 5b). This spatial alternation reinforces the central hypothesis of this study, as multi-peak potential profiles enhance localized membrane depolarization and lower excitation thresholds. Furthermore, aligned multi-contact stimulation focuses current delivery along superficial nerve paths, minimizing electrical dispersion into adjacent sensitive structures and potentially reducing overall current requirements [16].

To evaluate the effectiveness of the proposed EA in comparison to Cefaly electrode, all electrode EAs were individually generated and integrated with a head model to calculate the required stimulus current thresholds. As there are no standardized guidelines for determining the size of array electrode components, their dimensions and spacing are often chosen arbitrarily. However, it is important to recognize that overly small configurations might be ineffective for individuals with thicker fat layers, which can obstruct the current from reaching deeper tissue layers, such as nerve fibres. Additionally, smaller electrodes may result in high current densities, potentially causing discomfort and limiting the therapeutic efficacy of transcutaneous electrical stimulation (TES). Since electrodes serve as the interface between hardware and living tissue, their design must account for the geometry and characteristics of the target area. Circular electrodes are frequently used in biomedical applications because their smooth shape minimizes potential edge effects. To address these concerns, the electrode and metal contact were modelled as smooth circular shapes. The design of the EAs, including size and spacing, was based on variations in nerve trajectories, with the goal of identifying an effective configuration that satisfies all relevant criteria.

The Cefaly electrode was designed based on its dimensions (L × W = 94 mm × 30 mm), as shown in Figure 4a. The metallic contacts were assumed to be coated with a conductive hydrogel layer (Comfort Gel A hydrogel) to ensure stable and low-impedance contact with the skin. These self-adhesive electrodes were positioned on the forehead to stimulate branches of the frontal nerve [7] using biphasic current waveforms (see Figure 3). The proposed electrode thickness was designed based on the size of the Cefaly electrode.

To systematically evaluate the effect of contact sizing and spacing, five array configurations (EA1–EA5) were evaluated alongside the commercial Cefaly baseline. All geometric parameters, contact radii, inter-electrode spacings, polarity assignments, and channel delivery protocols are detailed in Table 1. In the proposed path-aligned design, the thickness of the electrode components was kept strictly constant across all configurations. Array variations were designated incrementally from EA1 to EA5 based on contact radii (r = 2.0 mm, 3.0 mm, 4.0 mm, 5.0 mm, and 6.0 mm), with inter-electrode edge-to-edge spacing (s) systematically increased to preserve geometric symmetry until reaching the defined boundaries of variation in the nerve trajectories. Electrode positioning was kept strictly constant (fixed) at standardized anatomical forehead coordinates across all simulation trials for both the proposed EAs and the baseline control.

Total injected current was increased dynamically based on the neural response to determine the 50% population recruitment threshold ($I_{50}$), with the exact same current ramping profile applied during evaluations of both Cefaly and the proposed configurations. Channel polarity assignments were configured following established dual-bipolar standards: for each EA, one electrode pair was designated as sourcing and the second pair sinking. As highlighted in Figure 4b, positive (+) and negative (−) polarities are explicitly color-coded in red and blue, respectively.

PA across all trajectory variations was evaluated across the five proposed geometries to identify the most effective contact dimensions for the defined nerve paths. Among the five tested configurations, EA2 (r = 3.0 mm, s = 2.5 mm) was identified as the best-performing configuration. Subsequently, the performance of EA2 was evaluated against the commercial Cefaly electrode control based on the current thresholds ($I_{50}$) required for nerve activation across 40 statistically generated nerve trajectories (10 stochastic variations per branch across SONs-I, SONM, SONL, and STN) embedded within one MRI-based realistic head model.

### 2.4. Finite Element (FE) Simulation

**Figure 6:**
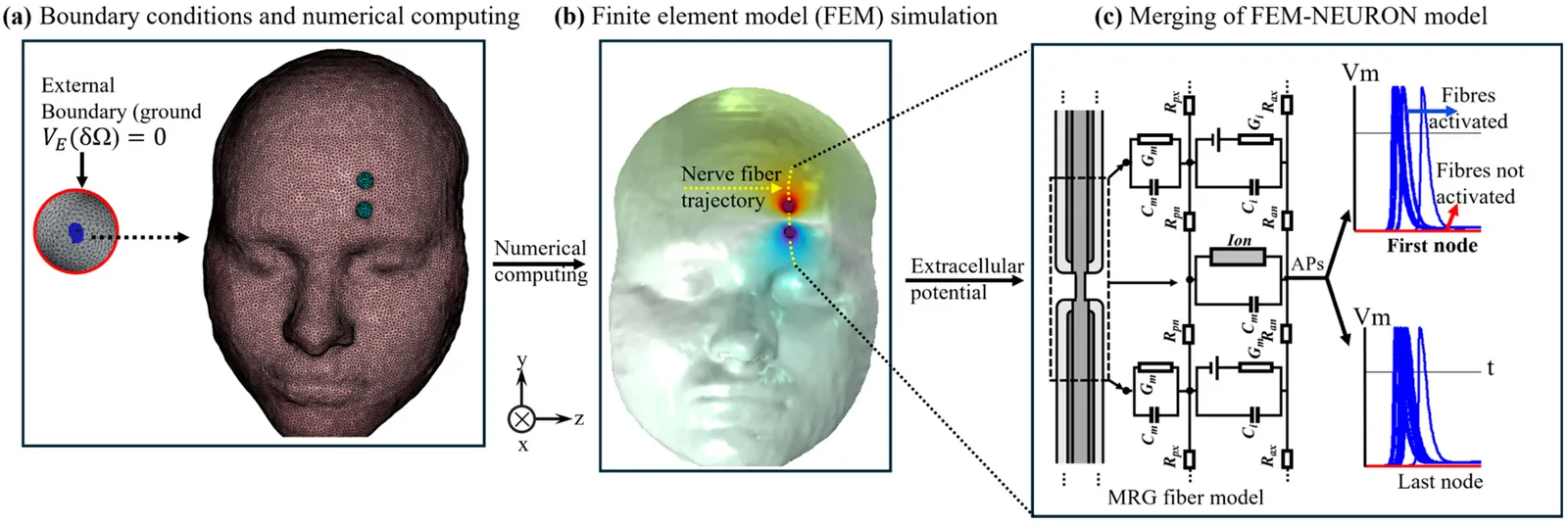
(**a**) The domains within the model were discretized. (**b**) The electrical potential variation across the nerve models were calculated. (**c**) To quantify percentage fiber activation (PA), electrical potential solutions from the finite element model (FEM) were coupled with NEURON simulations and subsequently imported into NEURON as extracellular stimulation inputs for myelinated fiber activation to calculate nerve activation. The activated nerve fibres are highlighted in blue, and the non-activated ones are highlighted in red.

The finite element method (FEM) was employed to calculate the electrical potential distribution within volume conductors by attaining the electrical properties of the tissue layers, as shown in Table 2. After defining the electrical properties of each layer, each model domain was discretized using tetrahedral finite elements to solve the partial differential equations numerically in COMSOL v5.2, as shown Figure 6a. The domains were meshed with finer discretization settings to optimal mesh quality without significantly increasing computational costs. Specifically, the electrode, skin, subcutaneous tissue, and muscle layers were meshed with a minimum element size of 1 µm and a relatively low growth rate of 1.2. In contrast, the remaining tissue layers were meshed using larger element sizes, ranging from 0.1 to 1 mm, to balance computational efficiency and accuracy.

Meshing the nerve model posed a challenge, as the nerve passes through multiple tissue layers. To address this, mesh settings for the nerve were adjusted, varying both the element size and growth rate depending on the specific model. For the outermost layer (sphere), which is distant from the region of interest, a larger element size was chosen, following standard tetrahedral mesh settings, as this layer had less influence on the neuroanatomical layers. The total number of finite elements used in the discretization process ranged from approximately seven to ten million, depending on the anatomical and nerve variations.

The TENS device approved for migraine therapy produces a biphasic, rectangular, and symmetric waveform characterized by a phase duration of 250 μs and operating at a stimulation frequency of 60 Hz [11]. At the onset of stimulation, the current intensity gradually increased over a period of 14 min until it reaches a peak value of 16 mA. Biological tissues exhibit both conductive (real) and reactive (permittivity-related) electrical properties. However, at relatively low frequencies (below 10 kHz), the conductive component predominates. Given that the TENS device functions at about 4 kHz, each domain in the models can reasonably be treated as purely conductive. Thus, the electrical conductivity values, as shown in Table 2, were assigned to calculate the simulation results (as shown in Figure 6b) using a quasi-static approximation of Maxwell’s Equations in COMSOL, which is expressed by the Laplace equation, as shown in Equation (4).

(4) $$\nabla \left(\left[\sigma \right]\nabla V_{FE}\right)=0$$

Here, σ denotes the low-frequency electrical conductivity of each tissue, assuming no frequency-dependent effects, and *V_FE_* represents the electric potential within the modeled geometry.

### 2.5. Myelinated Nerve Fiber Modelling

The excitation of nerve fibres via transcutaneous electrical stimulation (TES) was simulated using the McIntyre–Richardson–Grill (MRG) membrane model, which represents a myelinated mammalian axon [28]. The design of the compartments and their spatial arrangement along the nerve length was based on a double-layer cable model of a myelinated fiber, as described in [16]. Each compartment, representing passive properties between two active nodes, included two myelin attachment segments, two paranodal segments, and ten internodal segments.

Aβ fibres, with diameters (μD) following a Gaussian distribution with a mean of 12.5 µm and a standard deviation (σD) of 2 µm, were modeled according to experimental data from [29]. The relevant parameters for these fibers were derived by interpolating the experimental measurements from [16]. The length of each node was set to 1 µm. The first node of Ranvier was randomly positioned within the range of 0 to Δx along the nerve, where Δx denotes the node-to-node distance for a given fiber. Compartment insertion occurred between each pair of active nodes along the nerve’s trajectory, and the fiber model was terminated by a node at the end of the defined trajectory in the FEM model. This process was repeated for 100 nerve fibers in each nerve branch.

To construct a double-layer cable model for 100 fibres with imperfect myelin insulation, the geometric properties of the fibres, as well as the number and positions of the compartments along the nerve trajectory, were exported to NEURON [30]. The electrical properties of the ion channels were obtained from [28], while the node dynamics were extracted from the files in ModelDB, accession number 3810 [31].

### 2.6. Coupling FEM-NEURON Models

To quantify the percentage of fiber activations (PA), the electrical potential solutions from the FEM were integrated with NEURON models. The electrical potential along the nerve was computed in COMSOL, interpolated in MATLAB, and then imported into NEURON as extracellular potentials to stimulate the myelinated fibres, as shown in Figure 6c. In NEURON, the extracellular potentials were applied as symmetrical biphasic current pulses, each lasting 250 µs, with a repetition rate of 60 Hz, consistent with the protocol used in [11]. The amplitude of five consecutive current pulses was progressively increased until fiber activation was achieved, a process considered appropriate under the quasi-static approximation. Activation was confirmed when the fourth and fifth pulses produced identical responses, indicating stable nerve activation at the given stimulus. The PA was calculated for both the first and last nodes of the 100 fibres. A nerve was deemed to be activated when both the first and last nodes exhibited activation potential.

## 3. Results

The first step of this study was to determine the best-performing EA by analyzing the variation in nerve fiber percentage activations (PAs) as a function of the required stimulus current levels. This analysis was conducted using an anatomically realistic human head model in conjunction with average nerve models representing all branches (SONs-I, SONM, SONL, and STN). Subsequently, the best-performing EA was employed to evaluate the required stimulus current thresholds across all nerve model variations. Finally, the results were compared with those obtained using a Cefaly electrode model to assess the impact of the proposed EA on overall performance.

### 3.1. Effect of EA Size on Neural Activation

**Figure 7:**
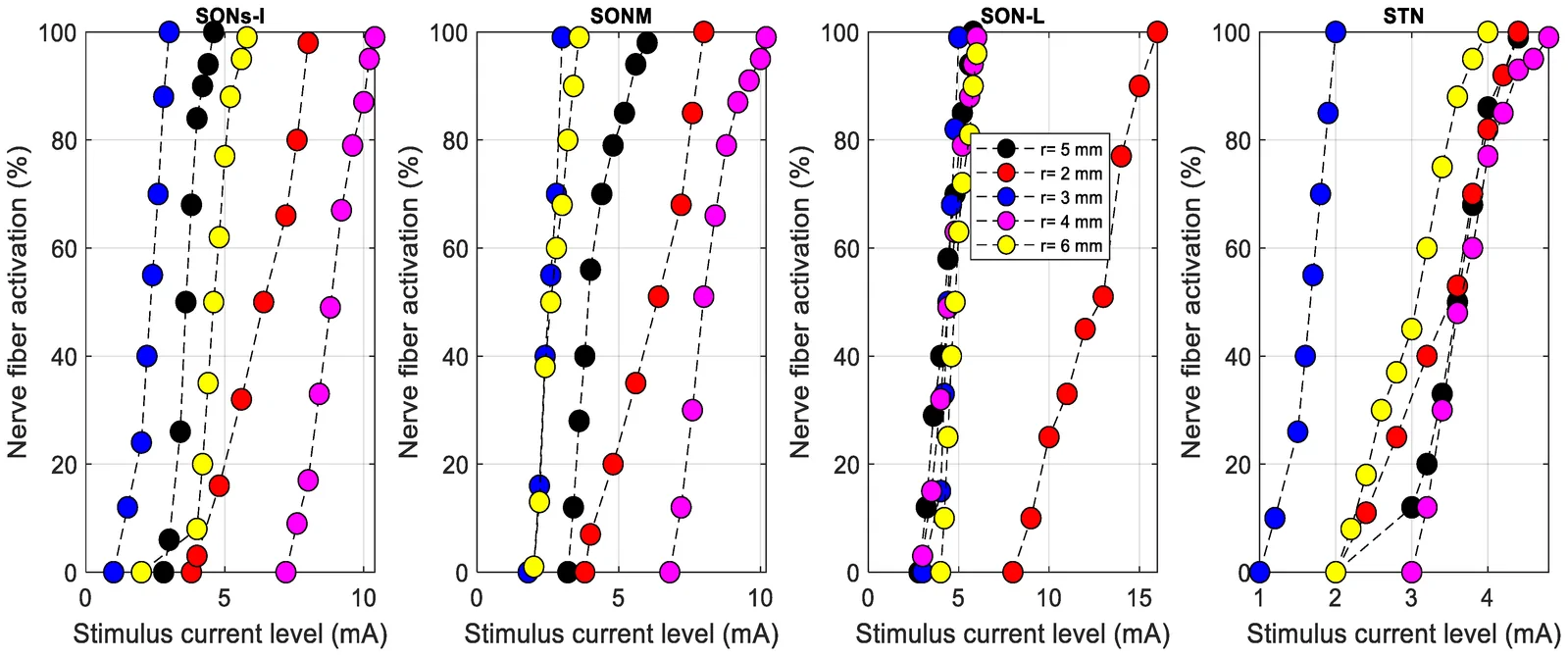
PA versus applied stimulation current (mA) for the SON-I, SON-L, SON-M, and STN nerve trajectories. Panels compare five electrode array configurations (EA1–EA5) with increasing contact radii: EA1 (r = 2 mm, red), EA2 (r = 3 mm, blue), EA3 (r = 4 mm, magenta), EA4 (r = 5 mm, black), and EA5 (r = 6 mm, yellow).

Five electrode array configurations (EA1 through EA5) with radii of 2 mm, 3 mm, 4 mm, 5 mm, and 6 mm were designed and simulated within an anatomically realistic human head volume conductor. Stimulus current levels were recorded across all configurations to identify the optimal array layout based on average nerve trajectories. Figure 7 illustrates PA as a function of 250 μs stimulus pulse amplitude along the supraorbital and supratrochlear nerve branches. The subplots further detail PA variations across the individual SON-I, SON-L, SON-M, and STN trajectories, and the resulting recruitment thresholds were analyzed to evaluate relative array performance.

Several key observations emerge from these data. First, the magnitude of the required stimulation current warrants emphasis: across all target branches, the current necessary to achieve complete fiber recruitment is substantially lower for the proposed arrays—particularly EA2—than values reported for conventional transcutaneous frontal nerve stimulators. However, threshold requirements vary across individual nerve branches. Higher current levels are generally required to activate fibers in the SON-L branch when using EA1. Notably, EA4 requires elevated current levels to recruit fibers across all nerve branches except SON-L. Consequently, EA1 and EA4 demand higher overall stimulation intensities, whereas the trends in Figure 7 demonstrate that EA2 represents the most efficient configuration. Specifically, EA2 consistently achieves target nerve activation at lower current levels than all other array layouts across all modelled trajectories.

### 3.2. Effect of Nerve Variations on Neural Activation

The best-performing array (EA2) was evaluated within an MRI-derived head model incorporating forty statistically generated nerve trajectories. Electric potential distributions produced by the Cefaly electrode and the proposed EA were solved using finite element analysis, interpolated in MATLAB, and imported into NEURON to simulate individual nerve fiber activation responses.

**Figure 8:**
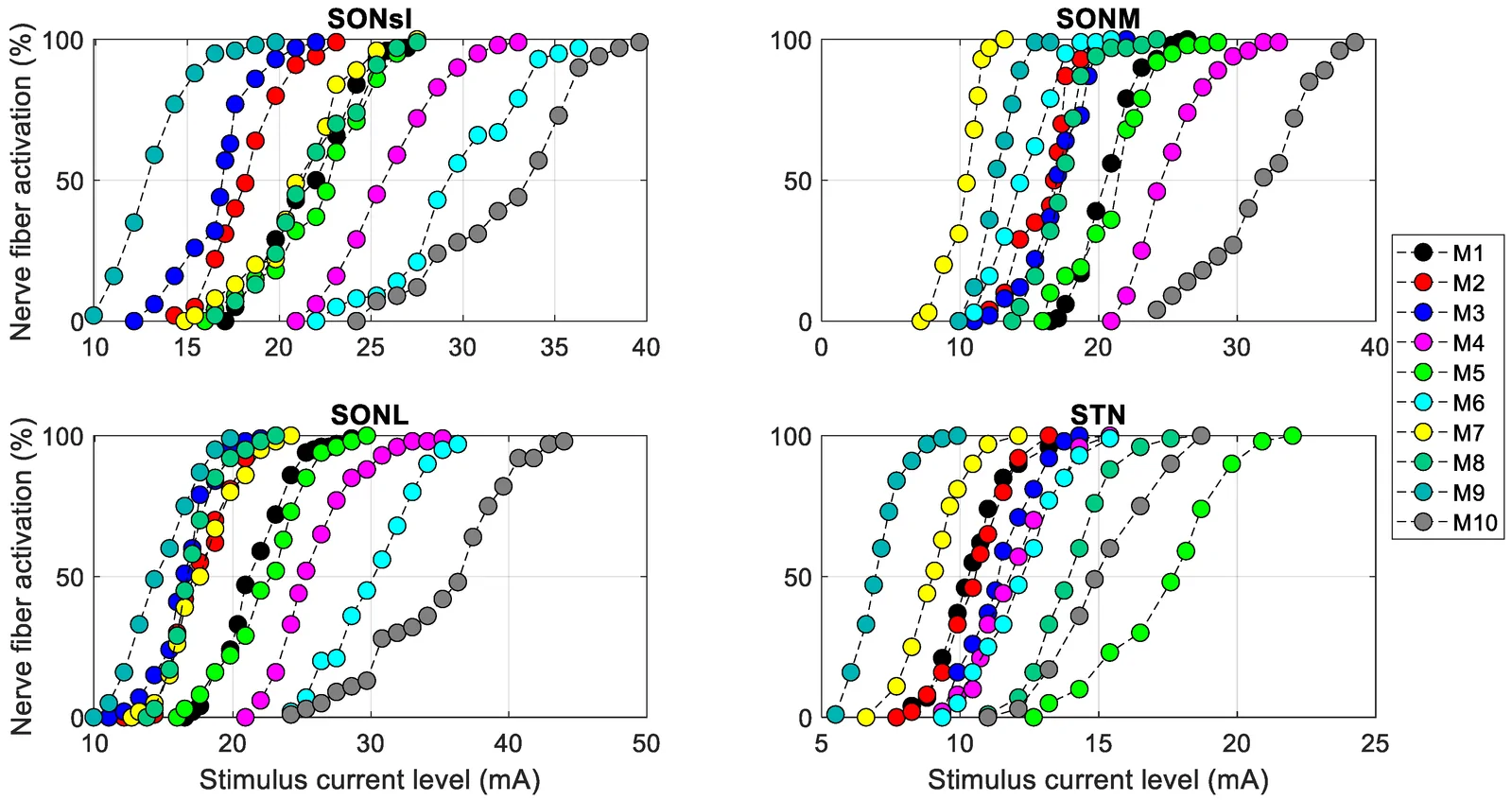
The relationship between PA and stimulation current amplitude is shown for different nerve trajectories across ten stochastic nerve trajectory populations using the Cefaly electrode. Variations in PA with respect to stimulus current are illustrated using different colours. M shows model number for each nerve branch.

Figure 8 presents the PAs as a function of the amplitude of 250 μs stimulus pulses for the 10 statistically relevant modelled (From M1 to M10) nerve cases using the Cefaly electrode for each nerve branch. Complete fiber recruitment (PA=100%) across the stochastic nerve trajectory models required stimulation currents ranging from 5 mA to 45 mA.

The relationship between required current levels and nerve activation varies across the different nerve branches. Among them, the SON-L branch consistently requires the highest stimulation currents, whereas the STN nerve fibres are activated at comparatively lower current levels. This pattern is observed in many models. Notably, model M10 exhibits the highest current requirements for the SON branches, a trend that does not extend to the STN fibres. In contrast, model M9 generally demonstrates lower current requirements across all nerve branches. In summary, the required current levels may be beyond the range of currents, which can be safely applied in this application.

**Figure 9:**
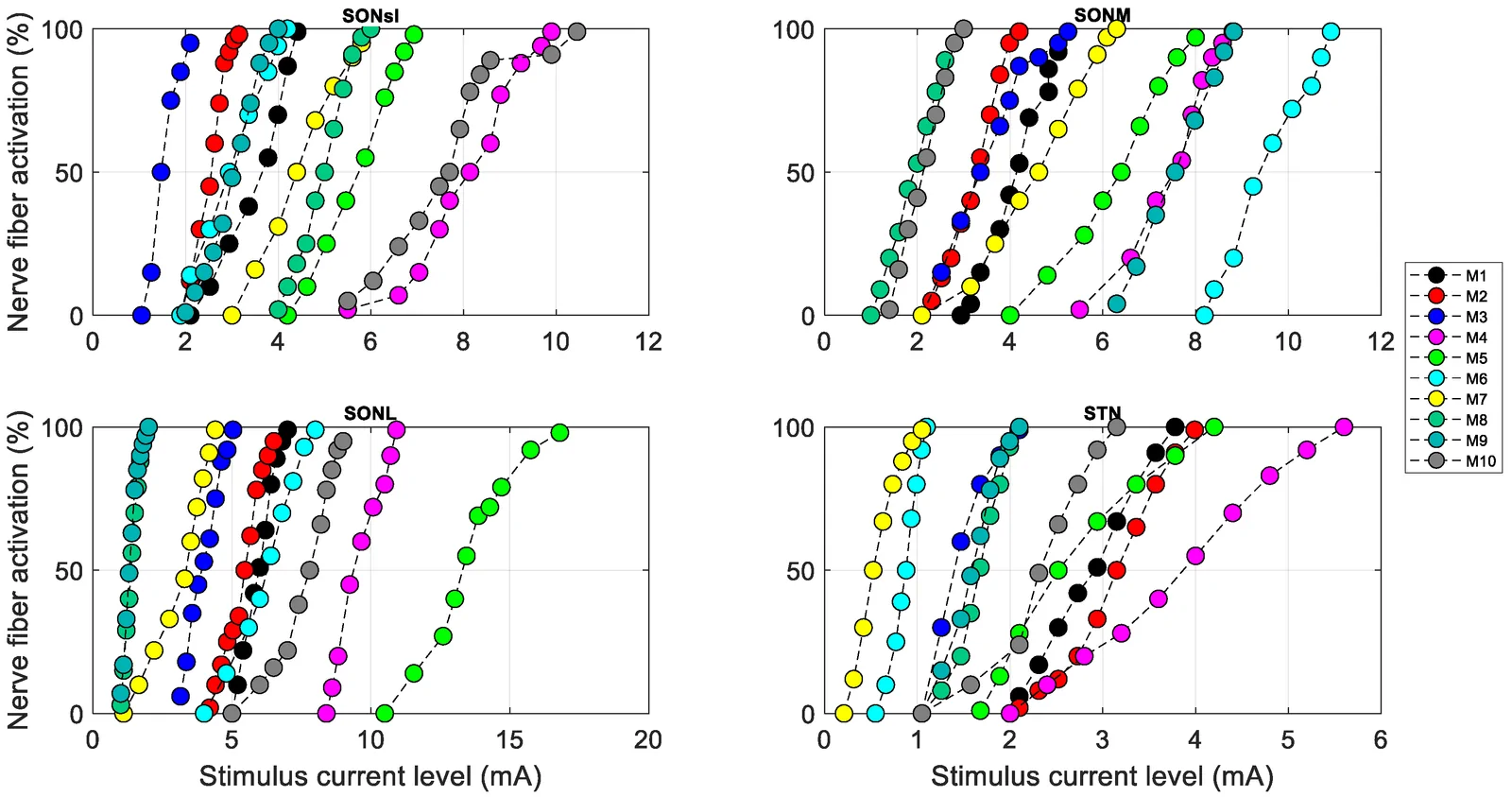
The relationship between PA and the required stimulation current amplitude is presented for different nerve trajectories across ten stochastic nerve trajectory populations using best-performing EA. Variations in PA as a function of stimulus current are depicted using different colors. M shows model number for each nerve branch.

Figure 9 shows the PAs as a function of 250 μs stimulus pulse amplitude for ten statistically representative nerve models (M1–M10) using the best-performing EA for each nerve branch. The results indicate that the majority of nerve models can be activated within a relatively low current range of approximately 1 mA to 10 mA (apart from Model5). Apart from the SONs–L fibres in model M5, fibres in the remaining nerve models generally require lower current levels to reach full (100%) activation. Furthermore, the stimulation thresholds for the SON branches are relatively similar to one another and are consistently higher than those required to activate the STN.

### 3.3. Comparison of Best-Performing EA with Cefaly Electrode

**Figure 10:**
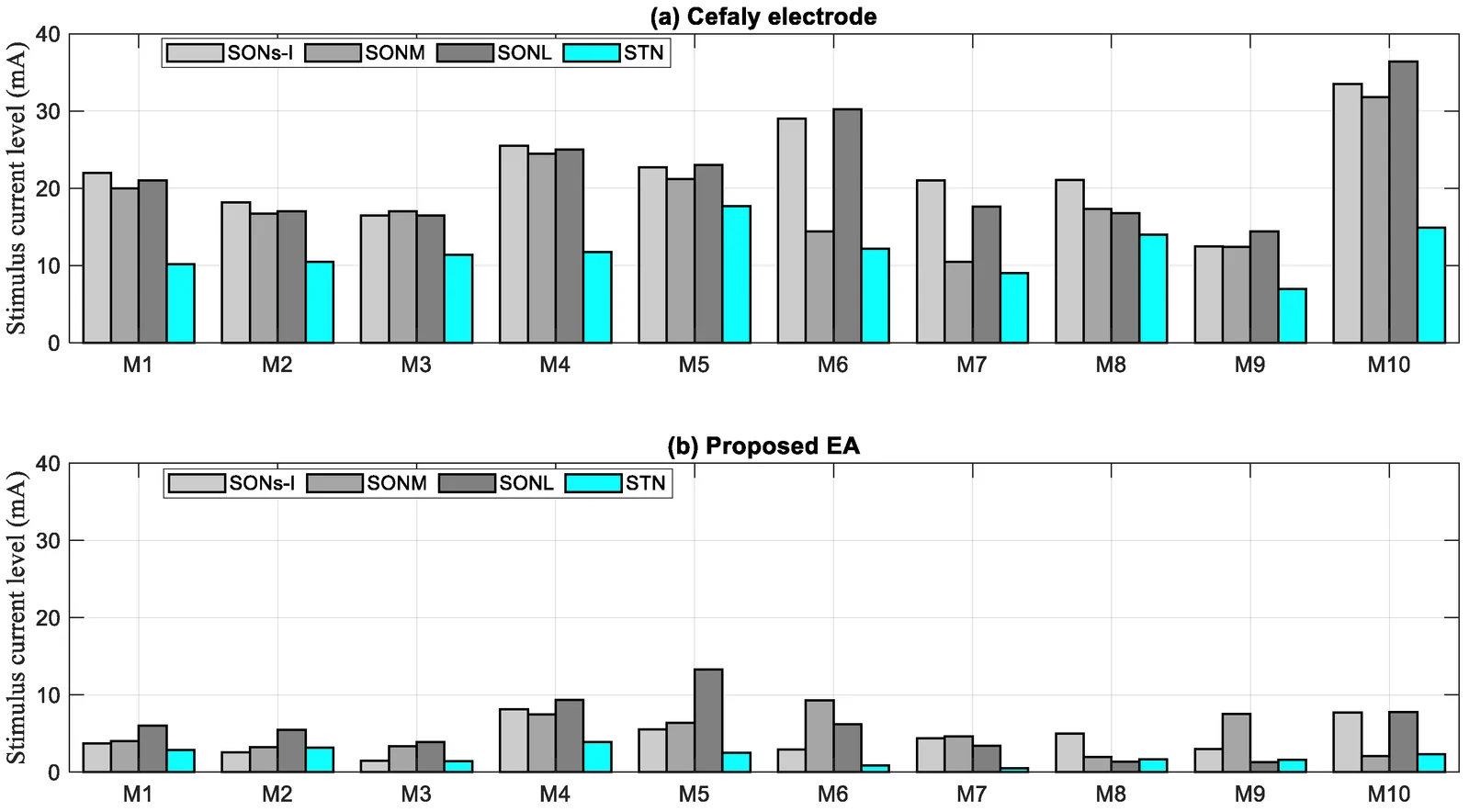
(**a**) Effect of the Cefaly electrode on the stimulation current required to achieve 50% fiber activation (I_50%_) across ten stochastic nerve trajectory populations (M1–M10). (**b**) Effect of the proposed EA on the stimulation current required for 50% fiber activation across the same ten nerve models. The nerve distributions represented in the bar plots are consistent with those shown in the previous figure. I_50%_ denotes the current amplitude required to activate 50% of the nerve fibres. Nerve branches are highlighted. M shows model number for each nerve branch.

Figure 10 compares stimulus current requirements (250 μs pulses) for the Cefaly electrode and the optimal EA2 array across ten stochastic nerve trajectory sets (M1–M10, 40 nerve models total). Based on evidence that 50% fiber recruitment ($I_{50}$) provides effective neural modulation [31], this threshold level was used to assess the impact of neuroanatomical variations across target branches. The bars in each subplot represent current thresholds for the STN, SON-M, SON-I, and SON-L branches, respectively. Compared to the Cefaly electrode, EA2 achieves a substantial reduction in recruitment thresholds across all stochastic populations. To recruit 50% of fibers across all branches, EA2 operates below 10 mA across nearly all models (with the sole exception of SON-L in set M5), whereas the Cefaly electrode requires at least 30 mA.

## 4. Discussion

Due to the target neuromodulator device being patient-operated, it may not be feasible to apply high current levels. Additionally, the electrode is positioned close to pain-sensitive structures in the current device, and even applying lower current levels may result in patient discomfort. Therefore, it is essential to optimize the existing electrode assembly to reduce the inefficiencies of the solution. It has been shown that aligning the electrode arrangements’ axis with the nerve trajectory can lower the required stimulus current levels. This alignment causes the polarity of the electrical potential along the nerve path to change based on the number of sources and sinks in the electrode configuration, as shown in Figure 5. The EAs system is a flexible approach that allows for dynamic adjustments in the size and positioning of stimulation electrodes to achieve an optimal design based on neural activation. However, using experimental methods to find the ideal electrode configuration is clearly time-consuming and impractical. Alternatively, finite element (FE) models that combine the human head, tissue layers, and nerve models can be employed to predict the best-performing electrode size and spacing between different EAs. In this study, such models were used to examine the influence of neuroanatomical variability and EAs on nerve fiber activation thresholds.

To ensure a statistically representative set of variations, potential differences in nerve trajectories by using various transition points from different anatomical layers were incorporated by sampling each parameter from its respective distribution, with sufficient separation between variables. Generating the complete model ensemble, which accounted for nerve variations across the anatomical layers of the human head, required over a day of computation using an automated MATLAB-based parameter generation script. In total, ten statistically representative nerve models were produced, covering forty models for all nerve branches. Together, these models encompass the full range of statistically significant anatomical variability considered in this study, enabling an investigation of how neuroanatomical variations and different electrode arrangements affect nerve activation thresholds. This comparison was made between the Cefaly electrode and the proposed best-performing EA.

The first phase of this study focused on identifying the best-performing array geometry by evaluating how PA varied with electrode contact size and spacing. PA and corresponding threshold current levels ($I_{50}$) were recorded across all stochastic nerve trajectories for each candidate array. The results demonstrated that EA2 (r = 3.0 mm, s = 7.0 mm) was the best-performing configuration across the targeted nerve branches, consistently requiring lower excitation thresholds compared to the other tested configurations.

These findings suggest that both smaller and larger array dimensions yield sub-optimal excitation efficiency within this volume conductor geometry. This response may be explained by the spatial characteristics of nerve activation, which is governed by the activating function (AF), defined as the second spatial derivative of the extracellular potential along the longitudinal nerve trajectory: $\mathrm{AF}=\frac{\partial^{2}V_{e}}{\partial x^{2}}$. To initiate an action potential, AF must exceed the local axonal membrane threshold across variable anatomical depth planes.

The reduced performance observed for configuration EA1 (r = 2.0 mm, s = 9.0 mm) may stem from its wide contact spacing, which spreads the injected current over a longer distance, potentially lowering the localized longitudinal potential gradient (AF) below threshold levels for smaller nerve ramifications. Conversely, as electrode contact radii increase and inter-electrode spacing narrows—reaching s = 1.0 mm in EA5—the extremely tight inter-electrode gap is hypothesized to cause superficial current shunting through low-impedance dermal layers. This shunting may result in a rapid field drop-off that fails to generate sufficient field penetration at deeper nerve trunks.

Configuration EA2 appears to achieve a favorable biophysical balance among the tested geometries: its geometric dimensions (r = 3.0 mm, s = 7.0 mm) and polarity alignment may maximize AF along longitudinal nerve pathways, facilitating adequate field penetration to deeper nerve branches without substantial dermal shunting while maintaining sufficient local current density to depolarize both superficial and lateral SON ramifications [32].

The results for the Cefaly electrode indicated that the stimulation currents required to activate the target nerves (STN and SON branches) range from 8 mA to 30 mA. In approximately 20% of the simulated cases, these thresholds exceed the maximum output capability of the Cefaly device (1–16 mA), meaning that effective stimulation cannot be achieved, which may limit device performance in these scenarios. Overall, the findings showed that a relatively high current range is generally necessary to activate the nerve fibres. In comparison with clinical findings, previous studies have reported an approximate 50% success rate in migraine reduction using the Cefaly electrode. Since the device is self-administered, discomfort or referred pain may occur even at stimulation levels below the device’s maximum output. This may reduce patient tolerance and adherence, ultimately affecting overall therapeutic effectiveness.

A key contribution of this study is the substantial reduction in activation current thresholds achieved by aligning the electrode axis with the target nerve pathways. The results showed that using proposed best-performing EA orientation significantly lowers the required stimulation currents, with approximately 95% of nerve models being activated at around 9 mA (apart from M5 for SONL). Importantly, all stimulation levels under this configuration fall within the operational limits of the existing device. In contrast, the Cefaly electrode typically requires at least 20 mA to activate the majority of nerve models. This indicates that the proposed EA reduces the stimulation current by at least 50%. At these lower current levels (approximately 9 mA), nerve activation may be achieved while minimizing the likelihood of pain induction, potentially improving patient comfort and tolerance.

**Figure 11:**
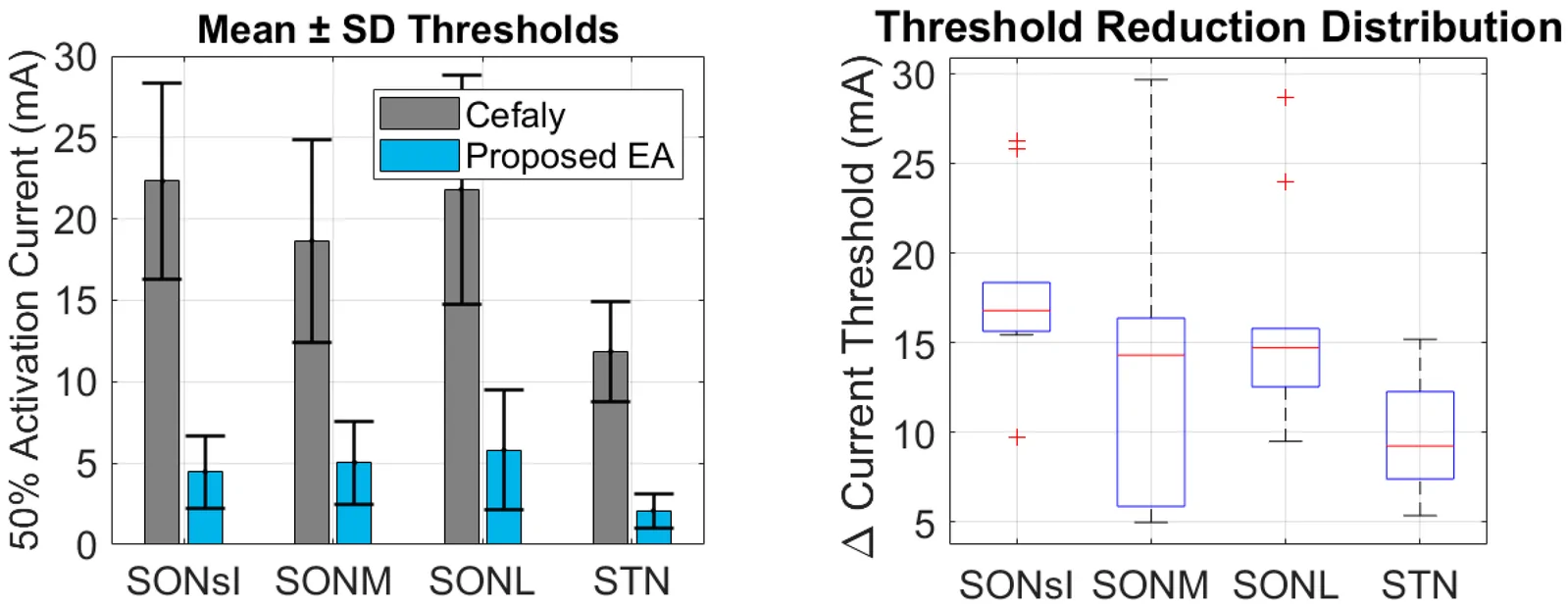
Statistical comparison of 50% nerve activation thresholds ($I_{50}$) between the Cefaly electrode and the proposed EA across 40 population nerve models.

Figure 10 demonstrated a marked reduction in current activation thresholds for the STN nerve fibres when proposed best-performing EA orientation is applied. Figure 11 shows a quantitative comparative analysis between the Cefaly electrode design and proposed EA. The proposed EA achieved a statistically significant reduction in the 50% nerve activation current threshold ($I_{50}$) across all four major target nerve branches. Specifically, the activation thresholds decreased from 22.32 ± 6.00 mA (Cefaly electrode) to 4.46 ± 2.20 mA (Proposed EA) for the SONs-I branch, and demonstrated similar consistent reductions in the SONM, SONL, and STN branches. The lower threshold variance (2.2) observed with the proposed design further indicates improved robust targeting despite head morphology differences. Overall, these findings confirm that the proposed EA substantially reduces the calculated activation thresholds of the overall current required to achieve targeted therapeutic activation.

Since nerve trajectories emerge from the skull and propagate through the subcutaneous tissue, their anatomical landmarks within these layers vary between individuals [26,32]. As a result, nerve fiber activation is influenced by both the anatomical configuration and the electrical properties of these tissues. Another important factor affecting the outcomes of this study is the spatial relationship between the nerve pathways and the stimulation of electrodes. In some cases, the SONs–L branch is located farther from the two electrode configurations, which typically results in higher required stimulation currents due to a reduced induced electric field compared with other nerve branches.

The best-performing array (EA2), aligned with the target nerve trajectory, produced lower activation thresholds across all stochastic variations compared to the Cefaly control. Notably, activating a single nerve branch may be sufficient for therapeutic migraine neuromodulation. In the EA2 configuration, the supratrochlear nerve (STN) and at least one supraorbital nerve (SON) branch were activated at calculated currents below 4 mA, indicating that lower inputs may achieve target fiber recruitment.

**Figure 12:**
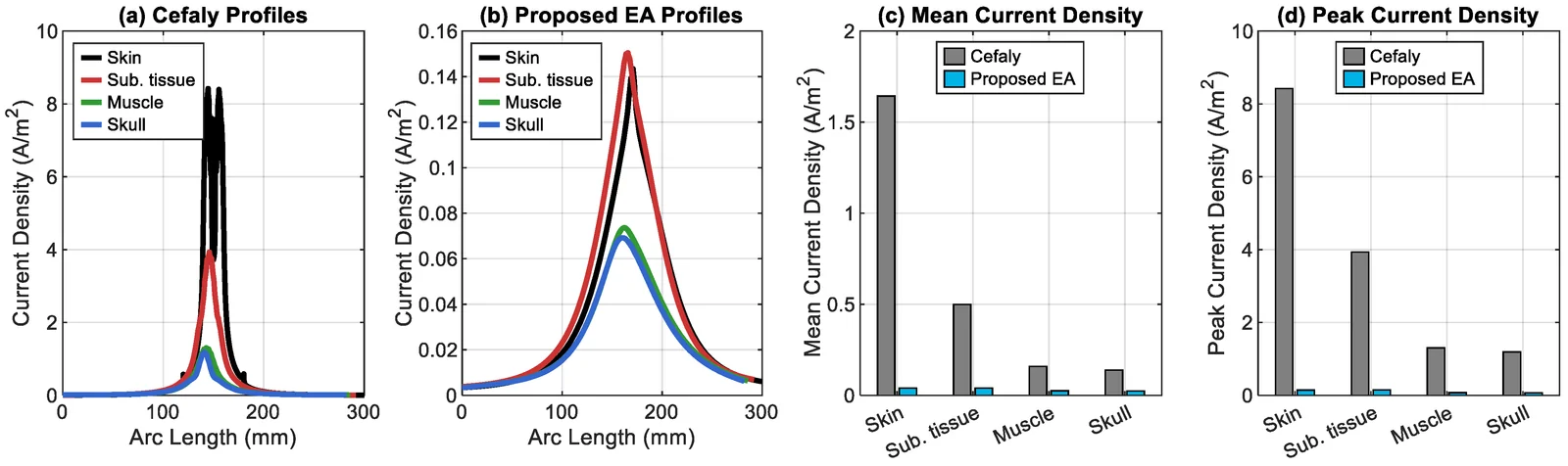
Spatial variations in current density (J, ${A/m}^{2}$) along the boundaries of sensitive anatomical layers for (**a**) the Cefaly electrode and (**b**) the proposed EA. (**c**) Mean current density and (**d**) peak current density evaluated across pain-sensitive tissue regions for both electrode configurations.

To evaluate field distribution near sensitive frontal structures, spatial current density profiles (J, in ${A/m}^{2}$) were extracted across superficial and deep layers (Figure 12). Current densities exceeding published reference thresholds (e.g., $0.6{\;A/m}^{2}$) have been linked to localized cutaneous irritation under forehead electrodes [33]. As shown in Figure 12, EA2 yields lower calculated peak current densities in cutaneous and subcutaneous layers relative to Cefaly by distributing surface current more uniformly across contacts.

Because low-intensity transcranial stimulation and transcutaneous frontal nerve stimulation (tFNS) share a transcutaneous delivery route, the $0.6{\;A/m}^{2}$ metric from Reference [33] serves as a useful benchmark for peak spatial current density. However, it does not represent a universal safety limit for pulsed tFNS. Under simulated conditions, EA2 demonstrates a potentially more favorable superficial field distribution; nevertheless, clinical safety, pain perception, comfort, adherence, and therapeutic efficacy require empirical and clinical validation.

Although the proposed electrode array (EA2) demonstrated lower calculated activation thresholds ($I_{50}$) across all simulated stochastic models, these computational findings must be interpreted within the context of several biophysical modeling assumptions. A primary limitation of this work is its sole reliance on computational finite-element and cable-neuron simulations, which necessarily simplify complex physiological phenomena. In this study, tissue layers were modeled using isotropic, purely resistive conductivities evaluated under quasi-static conditions, omitting frequency-dependent capacitive dispersion and structural anisotropy across dermal and muscular layers. Accordingly, the head tissue domain is accurately described as a multilayer conductivity model rather than a full dielectric dispersion framework.

Furthermore, the volume conductor model was constructed using a single MRI-derived head anatomy. Although target nerve paths were generated stochastically based on population-level statistical distributions anchored to primary facial landmarks (supraorbital notch, glabella, orbital rim), the model does not incorporate subject-specific nerve trajectories, individual variations in head shape or tissue layer thickness, or natural bilateral left-right cranial asymmetry. To maintain computational tractability, the neuroanatomical model accounts for the primary functional trunks (SON-I, SON-M, SON-L, and STN) but omits smaller terminal nerve branches, fine cutaneous arborizations, dermal nociceptor networks, microvasculature, and skin appendages.

From a translational perspective, real-world bioelectronic performance will depend on interface dynamics that were not explicitly simulated, including variable electrode–skin contact impedance, hydrogel thickness, contact pressure non-uniformity, mechanical skin deformation, adhesion stability, and skin condition variations during wear. Additionally, while path-aligned stimulation requires two independent channels—introducing modest hardware complexity compared to single-pad designs—practical alignment strategies, dynamic contact steering, and sensitivity to positioning or rotational errors were beyond the scope of this study. Consequently, the predicted activation thresholds and spatial current density profiles lack direct empirical validation, and comparative clinical trials will be necessary to determine whether these simulated biophysical distributions translate into clinical utility.

## 5. Conclusions and Future Remarks

This study established a multi-scale modeling framework integrating finite-element head volume conductors and compartmental MRG nerve models to assess how neuroanatomical diversity and electrode configuration influence transcutaneous frontal nerve stimulation thresholds. Results demonstrate that aligning electrode contacts along the principal nerve trajectory establishes alternating extracellular potential gradients, significantly reducing activation thresholds across all stochastic trajectory populations (SON-I, SON-M, SON-L, STN) compared to the baseline Cefaly electrode. The best-performing electrode array (EA2) substantially lowers required current levels while steering current density away from pain-sensitive superficial structures, potentially improving patient tolerance and therapeutic efficacy. Because these findings are computational, prospective clinical trials in human subjects remain necessary to confirm whether these threshold reductions translate into tangible improvements in pain relief and patient adherence in clinical practice. Future technical developments may leverage segmented fiber-shaped bioelectronics [34,35] to merge conformal wearability with multi-peak potential gradient steering.

## Figures and Tables

**Table 1:** Geometrical specifications and dimensions of the proposed path-aligned EA configurations.

Array ID	Contact Radius, *r* (mm)	Inter-Electrode Spacing, s (mm)
EA1	2	9
EA2	3	7
EA3	4	5
EA4	5	3
EA5	6	1

**Table 2:** The tissue layers and their conductivity.

Tissue Layers	Conductivity (S/m)	Source
Skin	0.22	[25]
Sub. tissue	0.025	[25]
Muscle (long.)	0.33	[25]
Muscle (trans.)	0.11	[25]
Nerve	0.083	[25]
Skull	0.015	[26]
Gray matter (Brain)	0.1	[26]
Eyeball	0.5	[25]
Sagittal sinuses	1.8	[27]
CSF	1.8	[27]
White matter	0.12	[25]

## Data Availability

The data supporting the findings of this manuscript are available from the corresponding author upon reasonable request.
